# Wavelength-Dependent Plasmon-Mediated Coalescence of Two Gold Nanorods

**DOI:** 10.1038/srep46095

**Published:** 2017-04-25

**Authors:** Jiunn-Woei Liaw, Wu-Chun Lin, Mao-Kuen Kuo

**Affiliations:** 1 Department of Mechanical Engineering, Chang Gung University, 259 Wen-Hwa 1st Rd., Kwei-Shan, Taoyuan 333, Taiwan; 2 Department of Mechanical Engineering, Ming Chi University of Technology, Taiwan; 3 Medical Physics Research Center, Institute for Radiological Research, Chang Gung University/Chang Gung Memorial Hospital, Taoyuan 333, Taiwan; 4 Center for Advanced Molecular Imaging and Translation, Chang Gung Memorial Hospital, Taiwan; 5 Institute of Applied Mechanics, National Taiwan University, 1 Sec. 4, Roosevelt Rd., Taipei 106, Taiwan

## Abstract

Plasmon-mediated coalescence of two nearby gold nanorods (NRs) suspended in water induced by the illumination of a linearly polarized (LP) light was studied theoretically. We analyzed the coupled optical forces and torques in terms of Maxwell’s stress tensor upon two identical NRs irradiated by a LP plane wave using the multiple multipole method to estimate the optomechanical outcome. Numerical results show that the light-matter interaction can perform attraction or repulsion, depending on their initial configurations. For the attraction, the end-to-end or side-by-side coalescence of the two gold NRs could be caused by the LP light, depending on the wavelength. For example, the side-by-side coalescence of two adjacent NRs of *r* = 15 nm and *L* = 120 nm is most likely induced by 800-nm LP laser beam, whereas the end-to-end coalescence by 1064-nm or 1700-nm LP laser. These distinct phenomena are attributed to the perpendicular or parallel alignment of NR to the polarization of LP light in different wavelength ranges. The magnitude of optical force, proportional to the light’s fluence, could be stronger than van der Waals force. The estimation based on quasi-static model without considering the fluid dynamics may provide an insight to optical manipulation on the self-assembly of gold colloid.

The growth mechanism of natural crystal has been studied for several centuries. A classical theory of Ostwald ripening was proposed first based on thermodynamics to explain this mechanism^1^; smaller particles tend to detach and diffuse toward another larger one spontaneously. Recently another theory of oriented attachment (OA) for the growth of nanocrystals has been recognized as the dominant mechanism for the process of colloidal self-assembly, whereby nearby nanoparticles can coalesce in a same crystal orientation to form a larger crystal^2^,^3^,^4^. The major driving forces of OA are the van der Waals force, dipole-dipole interaction, Coulombic force and so on^3^. Nevertheless since these driving forces are too weak, the natural OA process always takes a long time for nanoclusters to form micro-crystals. In the past decades, the formations of silver or gold anisotropic nanoparticles (NPs) induced by OA, e.g. nanorods (NRs) and nanowires (NWs), have been studied^4^. Because of the collective motion of free electrons corresponding to incident light, so-called the surface plasmon resonance (SPR), the light-induced polarizability of NPs is enhanced within the plasmon band of these metallic NPs. Hence, the optical properties of a variety of silver and gold NPs were studied extensively. In particular, the gold NRs have attracted a lot of attention due to the longitudinal SPR (LSPR), which is tunable positively depending on the aspect ratio (AR)^5^. Hence, various applications of gold NRs are being conducted to utilize the merit, particularly in near infrared regime^6^.

Furthermore, the coupled plasmons of multiple gold NRs in an end-to-end or side-by-side assembly perform versatile properties^7^,^8^,^9^,^10^. In the past decades, a variety of methods of using surface modification (e.g. polymer, ligand or DNA) for binding have been developed for the orderly self-assembly of NPs or NRs^7^. Recently, laser induced self-assembly of NPs or NRs has been studied^11^,^12^,^13^,^14^,^15^,^16^,^17^,^18^. In particular, ref. 16 has demonstrated the end-to-end assembly and welding of gold NRs caused by the irradiation of femtosecond laser. Additionally, ref. 18 has also experimentally shown that silver NRs can be assembled by the radially and azimuthally polarized CW lasers of 660 nm and 1064 nm with doughnut beam; the SEM images indicated the end-to-end coalescence of several silver NRs with an orientation parallel to the polarization of laser. In additional, the laser-induced chemical reduction of gold seeds and the subsequent laser-assisted OA for the growth of single-crystalline triangular gold nanoplates were studied^19^. The plasmon-mediated optical forces between two gold bipyramids or NRs have been investigated numerically^20^,^21^,^22^. In these studies the corresponding optical torque could play critical role in the alignment for these NPs’ coalescence^23^,^24^,^25^. The previous studies have proven that the optical torque induced by linearly polarized (LP) monochromatic light can cause the parallel or perpendicular alignment of a single silver/gold NR or nanowire *w.r.t.* the light’s polarization; the LSPR of a single NR is the turning point between the two modes^23^,^24^,^25^. Therefore, we are motivated to study the feasibility of the plasmon-mediated coalescence of two nearby NRs driven and guided by the optical force and torque, induced by a LP light. The multiple multipole (MMP) method is adopted for numerical simulation, which has been used for the analysis of the optical binding forces causing the long-range stable equilibrium between two gold or silver NPs as well as the optical torque on the alignment of a single NR^9^,^24^,^25^,^26^,^27^. In this paper, the optical forces and torques exerted on two coupled gold NRs in short-range interaction will be investigated through the surface integrations of Maxwell’s stress tensor on the surface of NRs in terms of the coupled electromagnetic (EM) fields^24^,^25^,^28^. Based on the optical forces and torques, we can estimate the most likely outcome of two mutually interacting gold NRs: repulsion or attraction; the latter could lead to the side-by-side or end-to-end coalescence, depending on the wavelength. The study of light-matter interaction of two coupled NRs with LP plane wave will provide an insight into the plasmon-mediated optomechanics on the self-assembly of NPs and the OA of elongated NRs or NWs under the irradiation of a continuous-wave (CW) LP laser beam.

## Results

**Figure 1:**
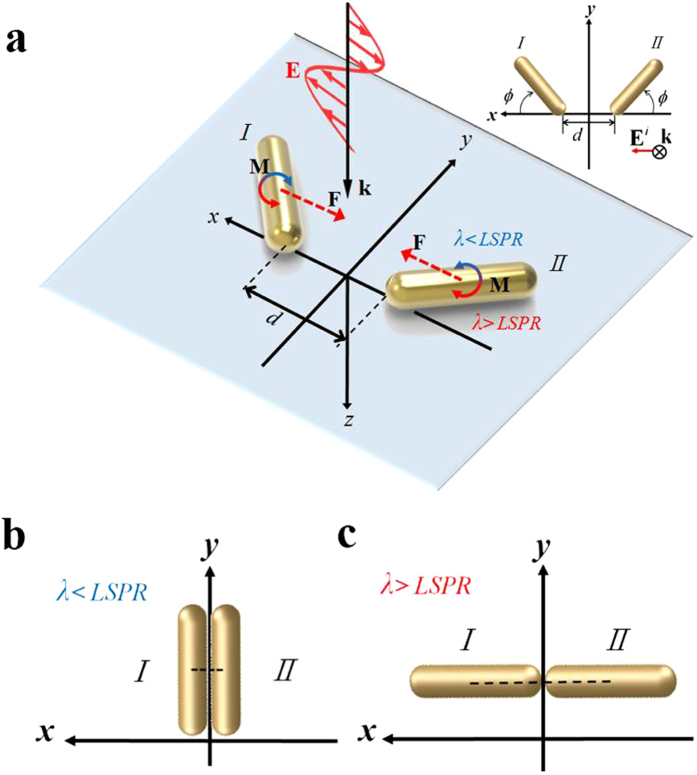
Configuration two coupled gold NRs irradiated by a normally incident LP light. (**a**) Configuration two coupled gold NRs (*I, II*) irradiated by a normally incident LP light with *x*-polarization. The final outcome of (**b**) the side-by-side coalescence due to attraction with perpendicular alignment or (**c**) the end-to-end coalescence due to attraction with parallel alignment.

**Figure 2:**
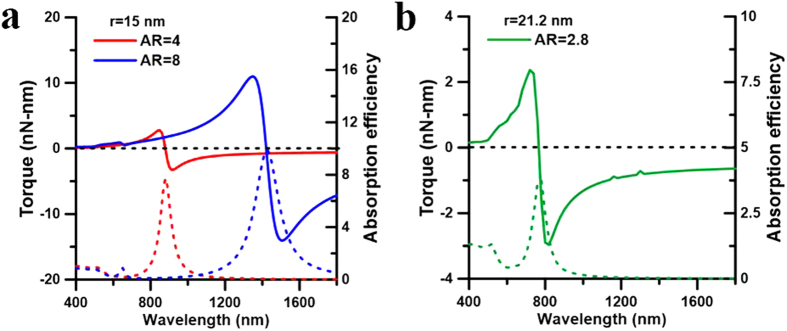
Spectra of optical torque and ACS efficiency of a single NR. The spectra of *z*-component optical torque and ACS efficiency on a single NR of (**a**) *r* = 15 nm with AR = 4 or 8, and (**b**) of *r* = 21.2 nm and AR = 2.8, where *ϕ* = 45°.

We assume that two adjacent gold NRs lying on the *xy* plane are irradiated by a normal-incident plane wave, propagating along the *z* axis (wavenumber vector: **k** = *k* **e**__*z*__) with *x*-polarization; the configuration is shown in Fig. 1(a), where the surrounding medium is water. The sizes of the two identical NRs are *r* = 15 nm and AR = 4. The MMP method was used to calculate the induced EM fields, where the irradiance (intensity) of plane wave is 25 MW/cm^2^. First, we analyze the optical-torque responses for the perpendicular and parallel modes of a single gold NR of *r* = 15 nm with AR = 4 or 8, and NR of *r* = 21.2 nm and AR = 2.8 at different wavelength regimes, where the angle between the long axis of NR and the light’s polarization is 45°, as shown in Fig. 2 23,24,25. Figure 2(a) shows that the spectra of *z*-component optical torque and absorption cross-section (ACS); the turning point between the positive and negative regimes of *z*-component optical torque upon a single NR of AR = 4 is at 880 nm, corresponding to the LSPR of a single NR^24^. This is to say that if the wavelength of a monochromatic light is shorter than the turning point the perpendicular alignment of a single NR *r.w.t.* the light’s polarization is induced by the optical torque. In contrast, if the light’s wavelength is longer than the turning point, the parallel alignment of a single NR is induced. If the two NRs is assumed to coalesce in the end-to-end manner to become an elongated NR of *r* = 15 nm and AR = 8, the LSPR of the newly formed NR is at 1420 nm, as shown in Fig. 2(a). On the other hand, if the two NRs is assumed to coalesce in the side-by-side manner to become a single thicker NR with *r* = 21.2 nm and AR = 2.8 (*L* = 120 nm), the LSPR of the new thicker NR is at 780 nm, as shown in Fig. 2(b). The above two wavelengths will be useful for us to estimate the subsequent optomechanical response of the newly formed NR after the end-to-end or side-by-side coalescence of two NRs of AR = 4 under the irradiation of different-wavelength LP light.

**Figure 3:**
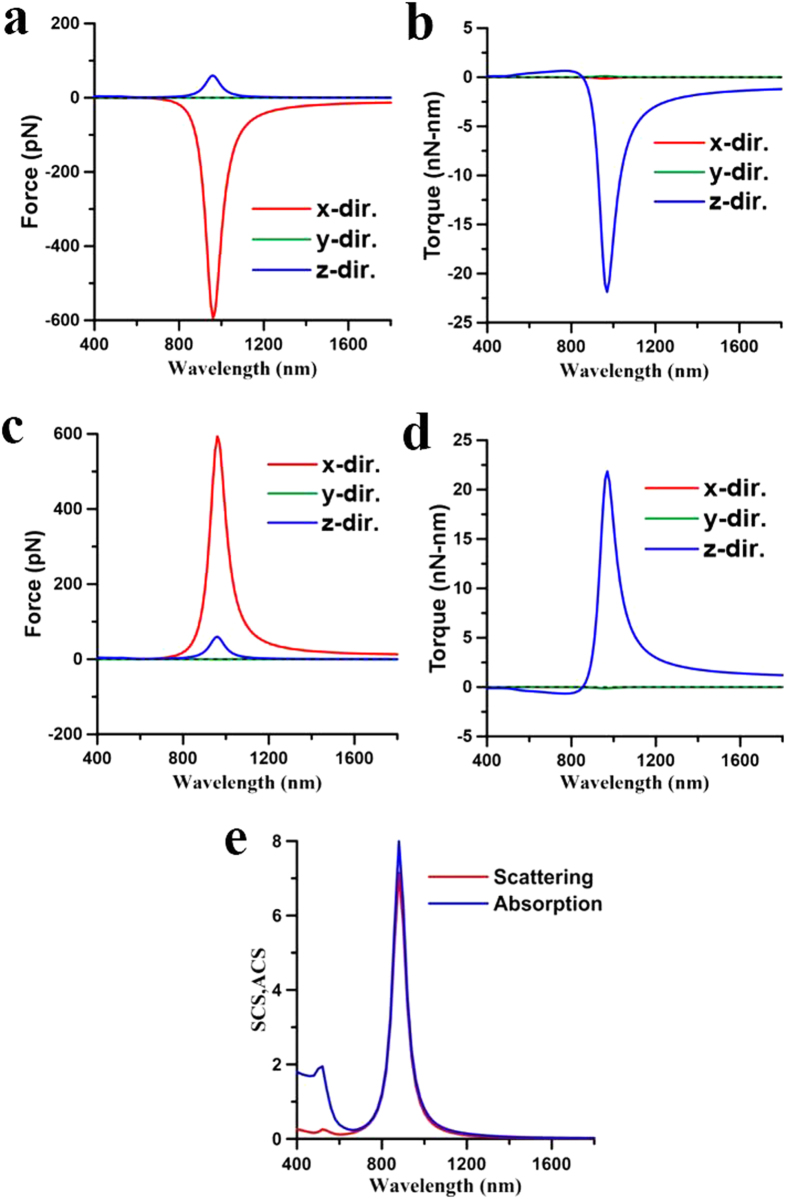
Spectra of optical forces and torques on two coupled NRs. The spectra of optical (**a**) forces and (**b**) torques on the NR of *I*, (**c**) forces and (**d**) torques on the NR of *II*, as well as (**e**) ACS and SCS efficiencies of the two coupled gold NRs with *ϕ* = 45° and *d* = 20 nm.

As for two plasmon-coupled NRs, the light-matter interaction becomes more complicated. In the following, several cases are discussed with spectrum analysis to identify the wavelength-dependent outcome. For example, the optical forces and torques upon the NRs of *I* and *II* versus wavelength are shown in Fig. 3, where the two NRs lie on the *xy* plane with typical postures of *ϕ* = 45° *r.w.t.* the *y* axis and a gap of *d* = 20 nm between their ends. Additionally, the spectra of scattering cross-section (SCS) and absorption cross-section (ACS) efficiencies of the two NRs with the above configurations also indicate that the coupled LSPR is at 880 nm, as shown in Fig. 3(e). The *z*-component optical-torque spectra demonstrate that there are two distinct regimes with different signs leading to different optomechanical responses for NRs: the perpendicular alignment in short-wavelength regime and the parallel alignment in the long-wavelength one. The turning point between the two regimes shown in Fig. 3(b) and (d) is at 865 nm, which is a little blue-shifted from that of coupled LSPR of two NRs (880 nm). If the wavelength of a LP monochromatic light is longer than 865 nm, the two NRs tend to be aligned parallel to the polarization (along *x* axis) of LP light by the *z*-component optical torques; 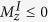 and 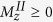. Moreover, the *x*-component optical forces exhibit the attraction; 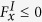 and 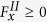. Here these optical forces and torques are exerted at the center of mass of each NR. As a result, the two NRs will coalesce with the end-to-end coalescence eventually; the schematic is shown in Fig. 1(b). In contrast, if the wavelength is shorter than 865 nm, they will be aligned perpendicular to the polarization of LP light by the *z*-component optical torques (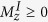, 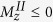) and also attracted by each other due to the attractive forces in the *x* direction (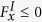, 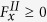); they eventually coalesce with the side-by-side coalescence, as shown in Fig. 1(b). We also found that the attractive forces and the aligning torques for the side-by-side coalescence in the short-wavelength regime are relatively weak compared to those for the end-to-end coalescence in the long-wavelength one. In fact, for the other configuration, e.g. the two NRs lying on the *xz* plane and on the both sides of *z* axis (see Fig. S1(a) in Supplementary Information), the results are similar to those in Fig. 3 (see Fig. S2 in Supplementary Information); the optical torques align them perpendicular to the polarization (*x* axis) and the optical forces perform the attraction. Consequently, the most likely outcome will also be the side-by-side coalescence.

On the other hand, the two adjacent NRs could repulse each other for certain initial configurations, resulting in no coalescence. For example, if they initially lie on the *xy* plane with special postures on the two sides of *x* axis (see Fig. S1(b) in Supplementary Information), the repulsive forces are observed in full spectra of *y*-component optical forces (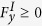, 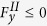), as shown in Fig. S3. Nevertheless, through the initial repulsion the two NRs will adjust their configurations and then perform the end-to-end or side-by side coalescence eventually; the most likely outcome, depending on the wavelength, is similar to the cases of Fig. 3.

These above results illustrate that the plasmon-mediated light-matter interactions of two coupling NRs depend on the wavelength of illumination as well as their initial postures. The optomechanical outcome of the two NRs could be repulsion or attraction, where the latter can result in the end-to-end or side-by-side coalescence. In order to illustrate the complicated plasmon-mediated interactions of two gold NRs in details, their optomechanical responses versus different initial postures at different wavelengths will be discussed individually. Three typical wavelengths are chosen for analysis; one is 800 nm shorter than the LSPR (880 nm) of a single NR of AR = 4, another 1700 nm longer than the LSPR (1420 nm) of an elongated NR of AR = 8, and the other 1064 nm in between the LSPRs of a NR (AR = 4) and an elongated one (AR = 8).

**Figure 4:**
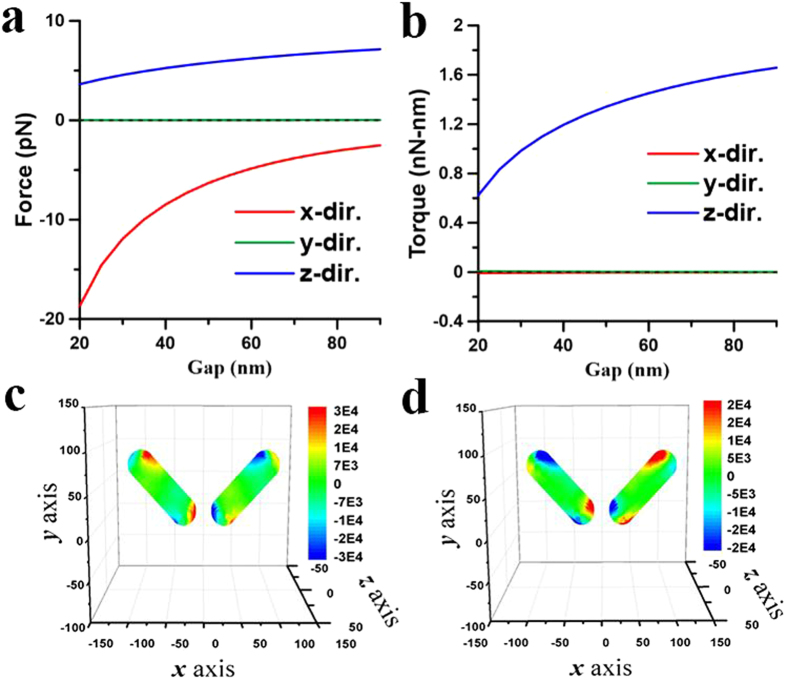
Optical forces and torques on two coupled NRs induced by 800-nm LP light leading to side-by-side coalescence. The optical (**a**) forces and (**b**) torques on the NR of *I* versus gap *d* at *λ* = 800 nm induced by an *x*-polarized light propagating along *z* axis. Both NRs lie on the *xy* plane and on the both sides of *y* axis with *ϕ* = 45°. The surface traction distributions of (**c**) **n** · **T** · **e**_*x*_ for the *x*-component force and (**d**) (**e**_*r*_ × **T** · **n**) · **e**_*z*_ for the *z*-component torque on two coupled NRs with *ϕ* = 45° and *d* = 40 nm. The outcome is the side-by-side coalescence.

**Figure 5:**
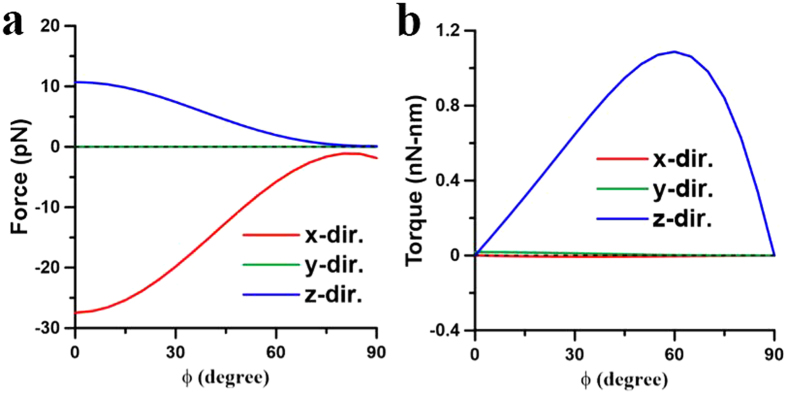
Optical forces and torques on two NRs with different angles induced by 800-nm LP light. The optical (**a**) forces and (**b**) torques on the NR of *I* versus angle *ϕ* at *λ* = 800 nm, where *d* = 20 nm. The outcome is the side-by-side coalescence.

**Figure 6:**
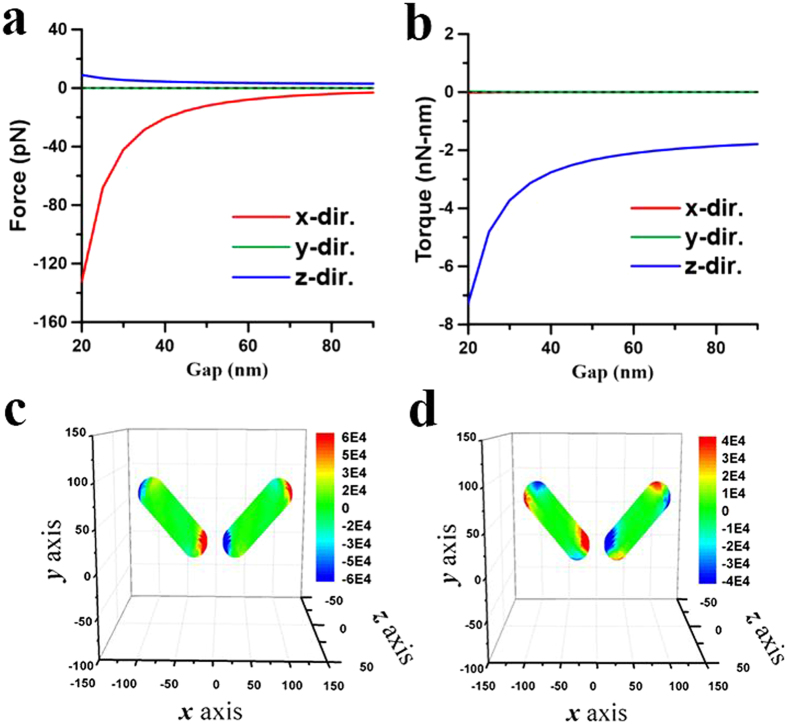
Optical forces and torques on two coupled NRs induced by 1064-nm LP light leading to end-to-end coalescence. The optical (**a**) forces and (**b**) torques on the NR of *I* versus gap *d* at *λ* = 1064 nm. The surface traction distributions of (**c**) **n** · **T** · **e**_*x*_ for the *x*-component force and (**d**) (**e**_*r*_ × **T** · **n**) · **e**_*z*_ for the *z*-component torque on two coupled NRs with *ϕ* = 45° and *d* = 40 nm induced by an *x*-polarized light propagating along *z* axis. The outcome is the end-to-end coalescence.

**Figure 7:**
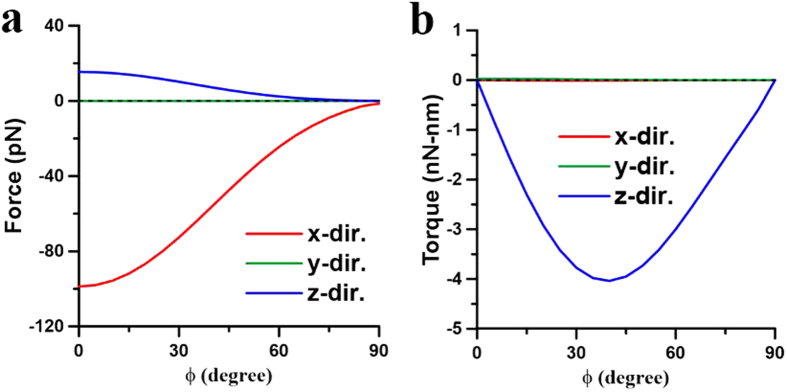
Optical forces and torques on two NRs with different angles induced by 1064-nm LP light. The optical (**a**) forces and (**b**) torques on the NR of *I* versus angle *ϕ* at *λ* = 1064 nm, where *d* = 20 nm. The outcome is the end-to-end coalescence.

**Figure 8:**
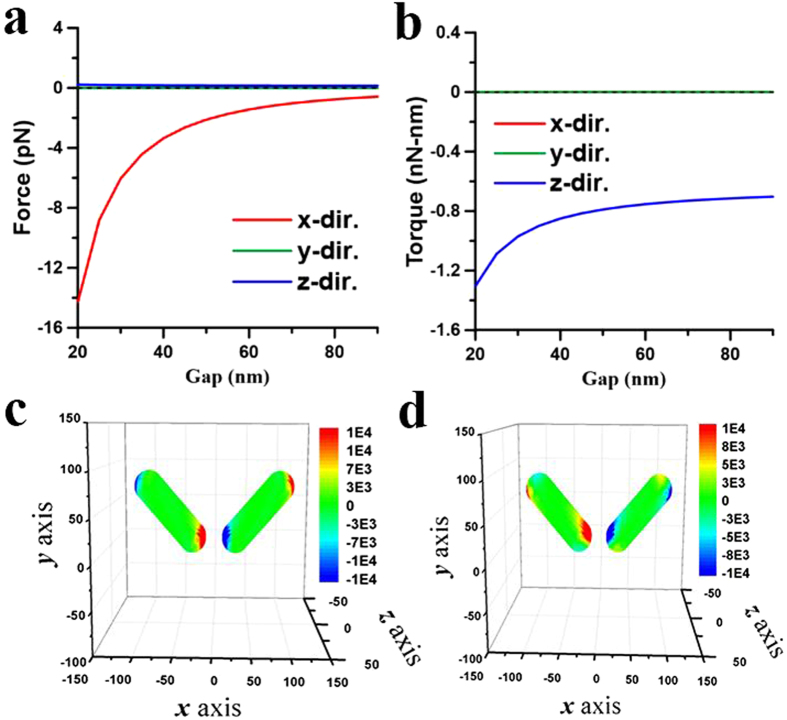
Optical forces and torques on two coupled NRs induced by 1700-nm LP light leading to end-to-end coalescence. The optical (**a**) forces and (**b**) torques on the NR of *I* versus gap *d* at *λ* = 1700 nm. Both NRs lie on the *xy* plane and on the both sides of *y* axis with *ϕ* = 45°. The surface traction distributions of (**c**) **n** · **T** · **e**_*x*_ for the *x*-component force and (**d**) (**e**_*r*_ × **T** · **n**) · **e**_*z*_ for the *z*-component torque on two coupled NRs with *ϕ* = 45° and *d* = 40 nm induced by an *x*-polarized light propagating along *z* axis. The outcome is the end-to-end coalescence.

**Figure 9:**
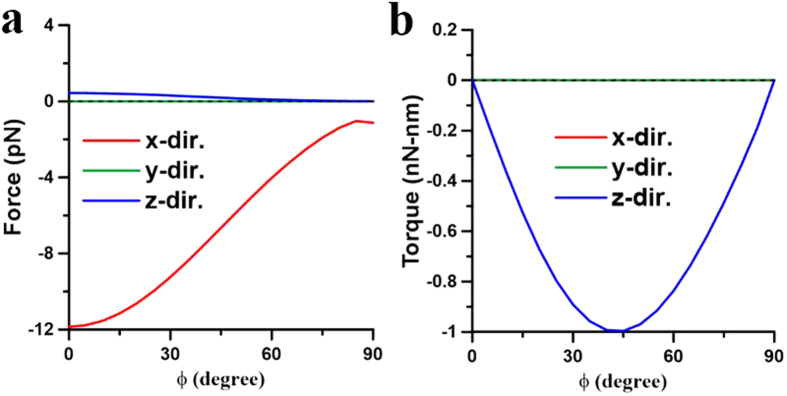
Optical forces and torques on two NRs with different angles induced by 1700-nm LP light. The optical (**a**) forces and (**b**) torques on the NR of *I* versus angle *ϕ* at *λ* = 1700 nm, where *d* = 20 nm. The outcome is the end-to-end coalescence.

1. *λ* = 800 nm. Since 800 nm is shorter than the LSPR (880 nm) of a single NR of AR = 4, a single NR performs the perpendicular alignment to the light’s polarization. For two nearby NRs with a gap of 20 nm, Fig. 3 has exhibited the side-by-side coalescence at 800 nm. Furthermore, Fig. 4(a) and (b) show the optical forces and torques upon the first NR against different gaps, where both NRs lie on the *xy* plane and on the both sides of *y* axis with *ϕ* = 45°. The results indicate that the *x*-component forces perform attraction (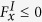, 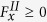) and the *z*-component torques drive NRs parallel to *y* axis (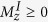, 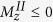); the smaller the gap the larger the attractive forces and torques are. Moreover, as the gap increases, the absolute values of optical torques decrease and approach to that on a single NR, which is non-zero. The surface traction distribution of **n** · **T** · **e**_*x*_ for the *x*-component force on these NRs is plotted in Fig. 4(c) to indicate the attractive behavior, where *d* = 40 nm and *ϕ* = 45°. Another surface traction distribution of (**e**_*r*_ × **T** · **n**) · **e**_*z*_ for the *z*-component torque is plotted in Fig. 4(d). The results show the *z*-component optical torques exerted on both NRs drive them in parallel to the *y* axis; both NRs tend to be perpendicular to the light’s polarization (*x* direction). Moreover, they attract one another because that the *x*-component optical forces exhibit the attraction. Additionally, Fig. 5 shows the optical forces and torques on the two NRs which lie on the *xy* plane and on the both sides of *y* axis with various angles, where the gap is 20 nm. From Figs 4 and 5, we can conclude that the eventual outcome performance of these NRs is most likely the side-by-side coalescence. Furthermore, once they coalesce and fuse together, an equivalent thicker NR of *r* = 21.2 nm and *L* = 120 nm is formed, whose LSPR is at 780 nm. Therefore, the final posture of the new thicker NR will change to the parallel mode as irradiated by LP laser beam of 800 nm. For two NRs with the other postures, the corresponding outcome also could be the side-by-side coalescence. For example, if the two NRs lie on the *xz* plane and on the both sides of *z* axis, the optomechanical responses of forces and torques also result in the side-by-side coalescence, as shown in Figs S4 and S5 (Supplementary Information). The above cases demonstrate that the most likely outcome of two adjacent NRs irradiated by a LP light of 800 nm is the side-by-side coalescence. The effective influence range of plasmon-mediated optical forces is roughly 100 nm, which positively depends on the intensity of laser beam. On the other hand, the two NRs could repulse each other in certain conditions. For example, if NRs lie on the *xy* plane and on the two sides of *x* axis initially, the *y*-component optical forces perform repulsion (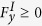, 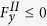), as shown in Figs S6 and S7 (Supplementary Information). Nevertheless, after the initial repulsion the two NRs will adjust their configurations and then perform the side-by-side coalescence eventually, which is the most likely outcome. According to our analysis, even though there is an offset between the centers of two NRs, the attractive forces make a center-to-center adjustment for the side-by-side coalescence.
2. *λ* = 1064 nm. Figure 6(a) and (b) show the optical forces and torques upon the first NR induced by 1064-nm laser versus gap *d*, respectively, where both NRs lie on the *xy* plane and on the both sides of *y* axis with *ϕ* = 45°. The *x*-component forces make them approach one another (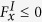, 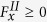), and the *z*-component torques align them in parallel to the polarization (*x* axis) (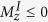, 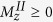); the smaller the gap the larger the attractive forces are. As the distance increases, the value of optical torque on two coupled NRs approaches that on single NR, depending on angle *r.w.t.* the polarization. For a special case of *d* = 40 nm and *ϕ* = 45°, the surface traction distributions of **n** · **T** · **e**_*x*_ for the *x*-component force and (**e**_*r*_ × **T** · **n**) · **e**_*z*_ for the *z*-component torque are shown in Fig. 6(c) and (d), respectively. The former shows the mutually attraction, and the latter shows the alignment in parallel to *x* axis. Consequently, the end-to-end coalescence of the two coupled NRs will be caused. Additionally, Fig. 7 shows the optical forces and torques on the two NRs, lying on the *xy* plane and on the both sides of *y* axis with a gap of 20 nm with various angles. Again, these results indicate that both NRs tend to be parallel to the light’s polarization by the optical torques and attracted by each other. As a result, the most likely outcome is the end-to-end coalescence due to the attractive forces in *x* direction. However, after the end-to-end coalescence, the new-formed NR of AR = 8 will be rotated by the optical torque to be perpendicular to the polarization of 1064-nm laser beam. This is because that for the LSPR of the elongated NR of *r* = 15 nm is at 1420 nm, which is longer than the incident wavelength of 1064 nm. For the two NRs with the other postures, the corresponding outcome also could be the end-to-end coalescence. For example, if the two NRs lie on the *xz* plane and on the both sides of *z* axis, the mechanical responses shown in Figs S8 and S9 (Supplementary Information) also result in the end-to-end coalescence. The above two cases demonstrate that the most likely outcome of two adjacent NRs irradiated by a LP light of 1064 nm is the end-to-end coalescence. On the other hand, the two NRs could repulse each other under special conditions. For example, if they lie on the *xy* plane and on the both sides of *x* axis, the optical forces of *y* component perform repulsion (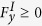, 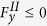), as shown in Figs S10 and S11 (Supplementary Information). Nevertheless, after the initial repulsion the two NRs will adjust their configurations and then perform the end-to-end coalescence eventually, which is the most likely outcome.
3. *λ* = 1700 nm. The optical forces and torques exerted on the *I*-NRs at *λ* = 1700 nm versus gap *d* are shown in Fig. 8(a) and (b), respectively, where the two NRs lying on the *xy* plane are on the both sides of *y* axis with *ϕ* = 45° irradiated by 1700-nm light. The surface traction distribution of 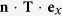 for the *x*-component force on these NRs is plotted in Fig. 8(c), where *d* = 40 nm and *ϕ* = 45°. Figure 8(a) indicates the attractive behavior (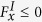, 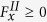). The surface traction distribution of (**e**_*r*_ × **T** · **n**) · **e**_*z*_ for the *z*-component torque is plotted in Fig. 8(d). Figure 8(b) and (d) show that the *z*-component torques drive NRs in parallel to the polarization of the incident light along *x* axis (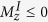, 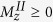). Additionally, Fig. 9 shows the optical forces and torques on two NRs on both sides of *y* axis versus angle *ϕ* with a gap of *d* = 20 nm. From Figs 8 and 9, we can also infer that the most likely outcome of two coupled NRs under the irradiation of 1700-nm LP light is the end-to-end coalescence, similar to that of 1064 nm. However, the forces and torques are relatively weak, compared with those induced by 1064-nm LP light. This is because that 1064 nm is closer to the LSPR (880 nm) of a single NR than 1700 nm. Moreover, after the end-to-end coalescence, the new elongated NR of AR = 8 still maintains parallel to the light’s polarization because its LSPR is at 1420 nm, which is still shorter than 1700 nm. This outcome induced by 1700-nm LP light is very different from that by 1064-nm light. For the other initial postures, the end-to-end coalescence of two NRs is also observed. For example, where the two NRs lie on the *xz* plane and on the both sides of *z* axis the attractive optical forces and corresponding torques make them tend to coalesce in the end-to-end manner, as shown in Figs S12 and S13 (Supplementary Information). On the other hand, the two NRs could repulse each other for certain configuration; e.g. Figs S14 and S15 (Supplementary Information) show that the coupled NRs will repulse each other (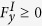, 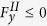) when they lie on the *xy* plane and are on the both sides of *x* axis (the polarization direction). Nevertheless, after the initial repulsion the two NRs will adjust their configurations and then perform the end-to-end coalescence eventually, which is the most likely outcome.

## Discussion

Numerical results illustrate that the end-to-end or side-by-side coalescence of two nearby and coupled NRs could be induced under the illumination of LP light, beside the repulsion. The optomechanical outcome depends on the wavelength of LP light *r.w.t.* the turning point (LSPR) of NRs as well as their initial postures. Our results indicate that the two NRs will be aligned parallel or perpendicular to the LP light’s polarization by the optical torque depending on the wavelength. In addition, due to the coupling optical forces the outcome could be repulsion or attraction depending on the initial configuration. The attractive optical forces associated with optical torques eventually can cause the end-to-end or side-by-side coalescence. For example, an 800-nm LP laser most likely induces the side-by-side coalescence, whereas a 1064-nm and 1700-nm LP lasers the end-to-end coalescence. For the former (800 nm), the wavelength is shorter than LSPR of a single NR; the optical torques make the long axis of each NR perpendicular to the polarization, and the coupled optical forces make them attract one another in the side-by-side manner. In contrast, for the latter two cases (1064 and 1700 nm), the wavelengths are longer than LSPR of a single NR; the optical torques align the axis of each NR parallel to the polarization, and the optical coupling forces make them attract one another in the end-to-end manner. Moreover, after the end-to-end coalescence at 1064 nm, the new elongated NR will be rotated to be perpendicular to the light’s polarization because of the wavelength of 1064 nm is shorter than the LSPR of the newly formed structure. In contrast, for 1700 nm, the new elongated NR will maintain parallel to the polarization.

The paper points out the possibility of light-driven end-to-end or side-by-side coalescence of two GNRs without the assistance of chemical affinity (e.g. ligand or functional group binding). In addition, the new driving force and torque, based on Maxwell’s stress tensor, are linearly proportional to the fluence of incident light, which could be stronger than the van der Waals force. This is of importance to the light manipulation on the self-assembly of gold colloid. According to ref. 29, the order of magnitude of the van der Waals force for the attraction of two gold spherical NPs of *r* = 50 nm with a distance 20 nm is about 0.1 pN. In contrast, the order of magnitude of plasmon-mediated optical force is about 10 pN as the intensity of light is 25 MW/cm^2^, two orders of magnitude larger than the van der Waals force. Moreover, the value of optical torque upon two coupled NRs approaches to that on single NR, which is a non-zero value, as the distance increases. For our cases, the order of magnitude of optical torque is around 1 nN-nm. In contrast, the magnitude of van der Waals torque is proportional to the van der Waals force, which decays rapidly as the distance increases. This is to say that if the intensity of light is strong enough the induced optical force and torque can help to accelerate the OA process of two coupled gold NRs. The plasmon-mediated light-matter interaction belongs to a short-range one of multiple NRs; the effective influence range positively depends on the intensity of laser beam, roughly more than 100 nm. The plasmon-mediated optical forces and torques exerted upon these NRs can play significant roles on their translational and rotational motions in aqueous suspensions to perform the coalescence in the end-to-end or side-by-side manner, if their values are larger enough to overcome the Brownian motion. In the plasmon-mediated coalescence process, the polarization of LP light provides the directionality for guiding the coalescence of two coupled NRs through the optical torques. If the wavelength of incident light is within the plasmon band, the plasmon-mediated polarizability of NRs can enhance the induced optical forces and torques to allow the colloidal self-assembly and coalescence possible. To analyze the dynamics of these NRs, we should consider the inertia force, Brownian motion, gravitation force, buoyancy and viscous drag force from surrounding medium (e.g. fluid), beside the optical forces. In particular, the Brown motion depending on temperature of water causes the stochastic perturbation on motion, and the drag force is a reactive force proportional to the relative motion (speed) of NRs to the fluid, where the viscosity is also dependent on the temperature. Hence, the dynamic analysis becomes more complicated. To clarify and identify the major role (driving force) of the optical force and torque playing on this light-driven coalescence, we therefore only studied the quasi-static case, where the relative motion of NRs to fluid is neglected, rather than the overall dynamic one, to estimate the possibility of light-driven end-to-end or side-by-side coalescence. Authors believe that utilizing the plasmon-mediated optical force and torque we can not only manipulate the self-assembly of gold/silver NPs and NRs but also accelerate the OA process of adjacent NRs.

## Method

In this paper, the MMP method was adopted to simulate the coupled EM fields (**E**, **H**) induced by the two NRs interacting with LP plane wave, as shown in Fig. 1 13,24,25,26. The MMP method is a semi-analytical method, where the expansion of different-ordered spherical wave functions at multi-centers is used to match the boundary continuity^27^. Subsequently, the optical forces and torques exerted on these NRs are analyzed. The plasmon-mediated optical force **F**^^*i*^^ and torque **M**^^*i*^^ exerted on the *i*-th (*i* = *I, II*) NR in terms of the EM fields are expressed as^24^,^25^


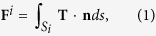



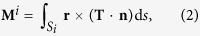


respectively. In Eqs (1) and (2), *S*__*i*__ is the surface of the *i-*th NR, **n** is the outward normal, and **r** is the position vector of any point on *S*__*i*__ with respect to the mass center of each NR. The average Maxwell’s stress tensor **T** of a period in terms of the total electric and magnetic fields (**E**, **H**) in the exterior zone is defined as^24^,^25^


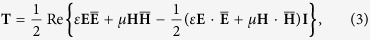


where Re denotes the real part, and the symbol of overbar the complex conjugate. In Eq. (3), **I** is the unit tensor of 3 × 3, as well as *ε* and *μ* are the permittivity and permeability of the surrounding medium, respectively. The total electric and magnetic fields outside the NRs are the linear combination of the incident and scattered fields, where the scattered field represents the coupled EM fields between the two NRs. In terms of Maxwell’s stress tensor **T**, the surface traction vectors **T** · **n** and **e**_*r*_ × **T** · **n** on the surfaces of NRs will be analyzed to illustrate the effects of the optical force and torque, respectively. Additionally, the frequency-dependent permittivity of gold presented in ref. 30 is used for the MMP simulation. From the optical forces and torques, we can estimate the light-driven outcome of the two NRs.

## Additional Information

**How to cite this article:** Liaw, J.-W. *et al*. Wavelength-Dependent Plasmon-Mediated Coalescence of Two Gold Nanorods. *Sci. Rep.* **7**, 46095; doi: 10.1038/srep46095 (2017).

**Publisher's note:** Springer Nature remains neutral with regard to jurisdictional claims in published maps and institutional affiliations.

## Supplementary Material

Supplementary Information
