# Supplementary material for: Wavelength-Dependent Plasmon-Mediated Coalescence of Two Gold Nanorods
Source: Sci Rep. 2017 Apr 25;7:46095. doi: 10.1038/srep46095 (PMC5404630; doi:10.1038/srep46095)
Supplement: Supplementary Information [file srep46095-s1.pdf]

# Wavelength-Dependent Plasmon-Mediated Coalescence of Two Gold Nanorods

Jiunn-Woei Liaw[1,2,3,4*], Wu-Chun Lin[5], and Mao-Kuen Kuo[5*]
[1]Department of Mechanical Engineering, Chang Gung University, 259 Wen-Hwa 1st Rd., Kwei-Shan, Taoyuan 333, Taiwan
[2]Department of Mechanical Engineering, Ming Chi University of Technology, Taiwan
[3]Medical Physics Research Center, Institute for Radiological Research, Chang Gung University/Chang Gung Memorial Hospital, Taoyuan 333, Taiwan
[4]Center for Advanced Molecular Imaging and Translation, Chang Gung Memorial Hospital, Taiwan
[5]Institute of Applied Mechanics, National Taiwan University, 1 Sec. 4, Roosevelt Rd., Taipei 106, Taiwan
*Corresponding authors: markliaw@mail.cgu.edu.tw, mkkuo@ntu.edu.tw

The wavelength-dependent mechanical responses of two identical and adjacent gold nanorods (NRs) (*I*, *II*) of *r*= 15 nm and AR= 4, emerged in water irradiated by an *x*-polarized plane waves propagating along the *z* axis, are studied by using MMP method. The intensity of incident plane wave is 25 MW/cm$^2$. Two configurations of the two coupled NRs are analyzed in the following, as shown in Fig. S1: the NRs lying on (a) the *xz* plane and on the both sides of *z* axis with a gap *d* and an angle $\theta$, and on (b) the *xy* plane and on the both sides of *x* axis with a gap *d* and an angle $\phi$.

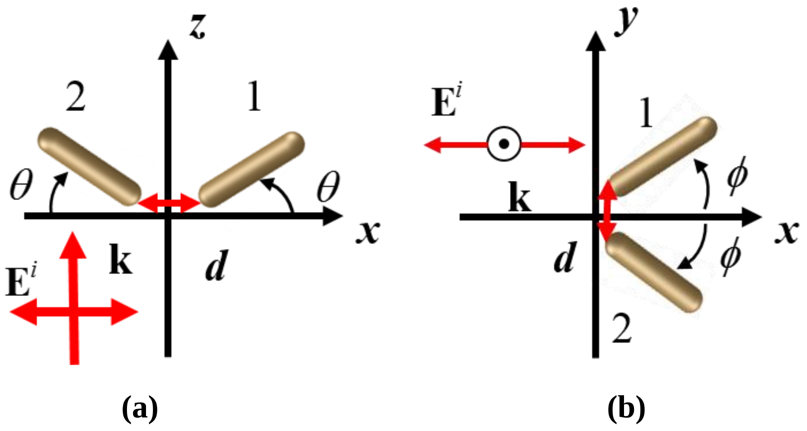


**Figure S1.** Configuration of two coupled NRs lying on (a) the *xz* plane and on the both sides of *z* axis with a gap *d* and an angle $\theta$, or on (b) the *xy* plane and on the both

sides of $x$ axis with a gap $d$ and an angle $\phi$ irradiated by $x$-polarized plane wave propagating along the $z$ axis.

For the case of Fig. S1(a) with $\theta = 45^{o}$, the $x$-component optical forces on both NRs perform the attraction in the full spectrum ($F_x^I \leq 0$, $F_x^{II} \geq 0$), as shown in Fig. S2, and the $y$-component optical torques exhibit the wavelength-dependent behavior again: the perpendicular and parallel modes. For this configuration, the two NRs will be aligned perpendicular to the polarization ($x$ axis) ($M_y^I \leq 0$, $M_y^{II} \geq 0$, if $\lambda < 865$ nm) or parallel to the polarization ($M_y^I \geq 0$, $M_y^{II} \leq 0$, if $\lambda > 865$ nm); the former is the perpendicular mode, and the latter the parallel mode. The turning point between the two regimes is at 865 nm, which is very similar to Fig. 3. Eventually, the most likely outcome in the two regimes will be the side-by-side ($\lambda < 865$ nm) or the end-to-end ($\lambda > 865$ nm) coalescence, respectively.

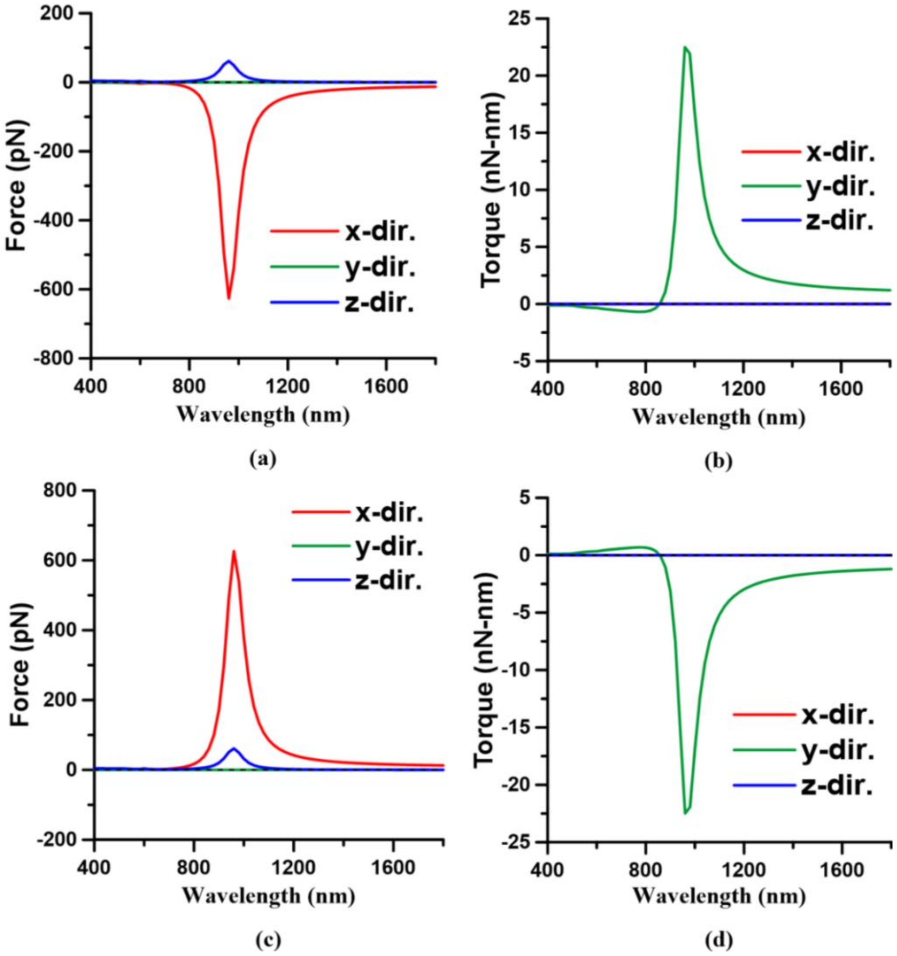


**Figure S2.** The spectra of optical (a) forces and (b) torques on the NR of *I*, and (c)

forces and (d) torques on the NR of *II* for the case of Fig. S1(a) with $\theta$= 45$^o$ and $d$= 20 nm.

On the other hand, for the case of Fig. S1(b) with $\phi$= 45$^o$ and $d$= 20 nm, the two adjacent NRs could repulse each other, as shown in Fig. S3. The repulsive forces are observed in the full spectra of *y*-component optical forces ( $F_y^I \geq 0$ , $F_y^{II} \leq 0$ ), resulting in no coalescence.

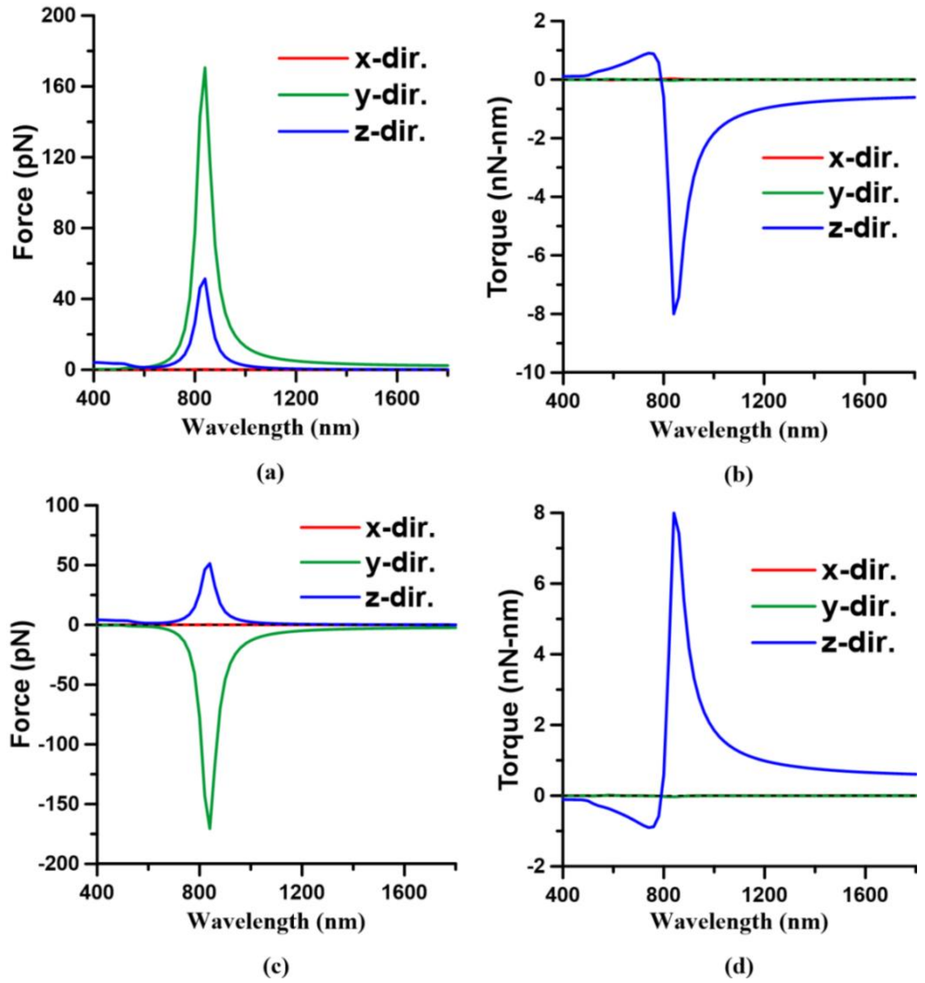


**Figure S3.** The spectra of optical (a) forces and (b) torques on the NR of *I*, as well as (c) forces and (d) torques on the NR of *II* for the case of Fig. S1(b) with $\phi$= 45$^o$ and $d$= 20 nm.

For the case of Fig. S1(a) at $\lambda$= 800 nm, the optical forces and torques exerted on both NRs are shown in Figs. S4 and S5. The *y*-component optical torques drive these NRs parallel to the *z* axis (perpendicular mode), and the *x*-component optical forces perform the attraction. As the gap decreases, the attractive forces increase. Consequently, the two NRs can coalesce with one another in the side-by-side manner for this initial configuration.

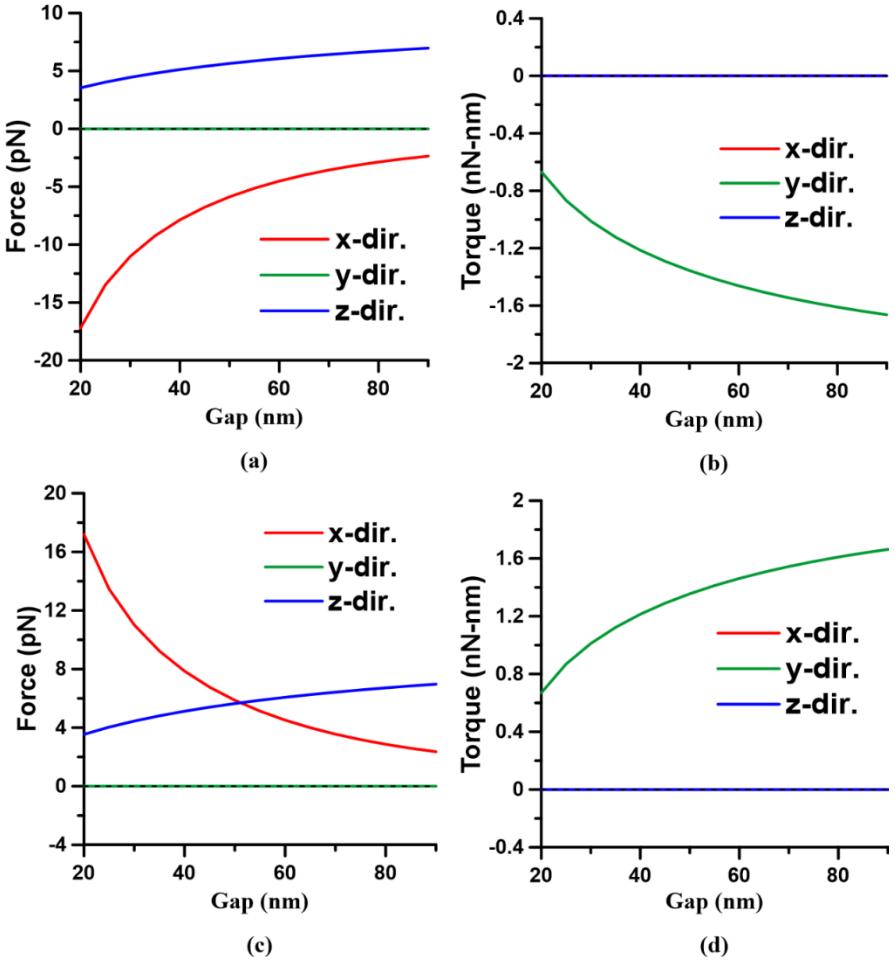


**Figure S4.** The optical (a) forces and (b) torques on the NR of *I* versus gap *d*, and (c) forces and (d) torques on the NR of *II* for the case of Fig. S1(a) with $\theta$= 45$^{o}$ at $\lambda$= 800 nm. The outcome is the side-by side coalescence.

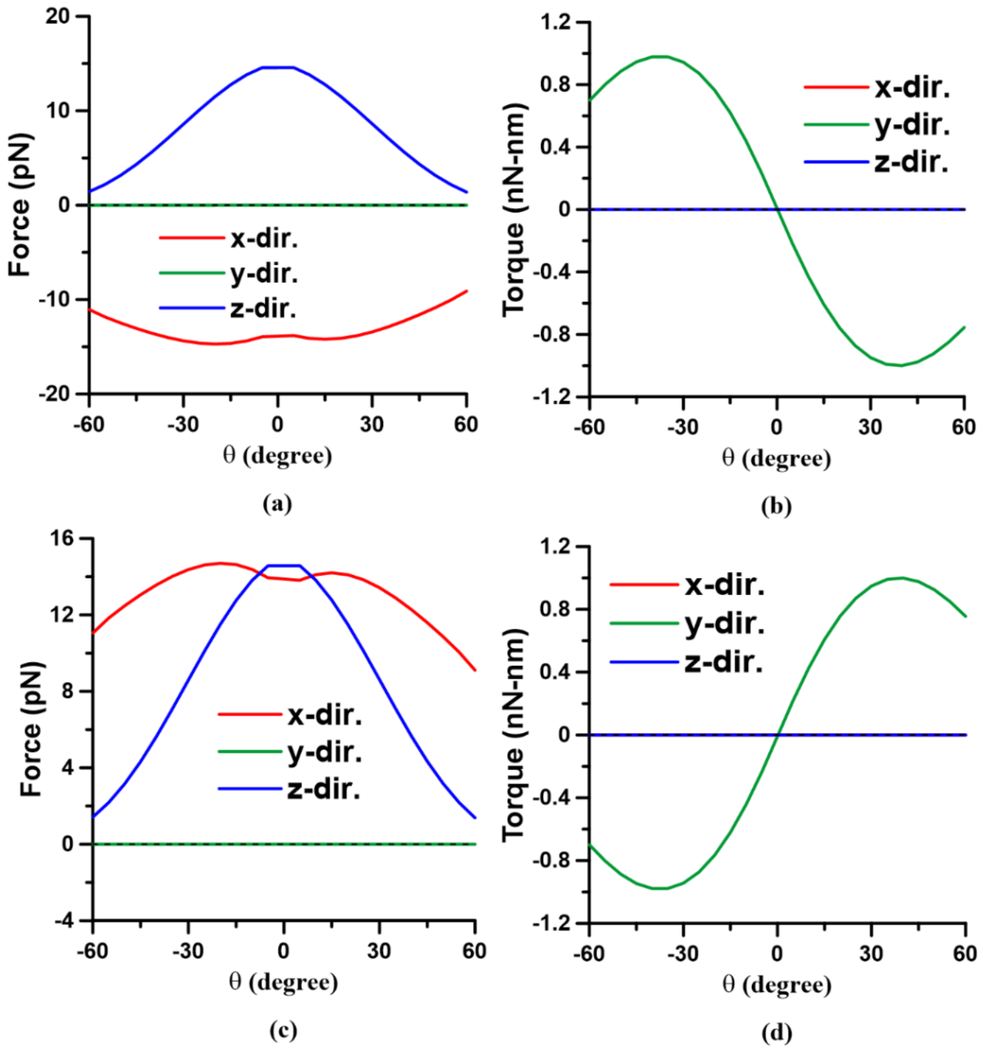


**Figure S5.** The optical (a) forces and (b) torques on the NR of *I* versus angle $\theta$, and (c) forces and (d) torques on the NR of *II* for the case of Fig. S1(a) with $d$= 20 nm at $\lambda$= 800 nm. The outcome is the side-by-side coalescence.

For the case of Fig. S1(b) at $\lambda$= 800 nm, the optical forces of $y$ component on both NRs, as shown in Figs. S6 and S7, perform the repulsion ($F_y^I \geq 0$, $F_y^{II} \leq 0$). Hence, the two NRs cannot coalesce with one another for the initial postures.

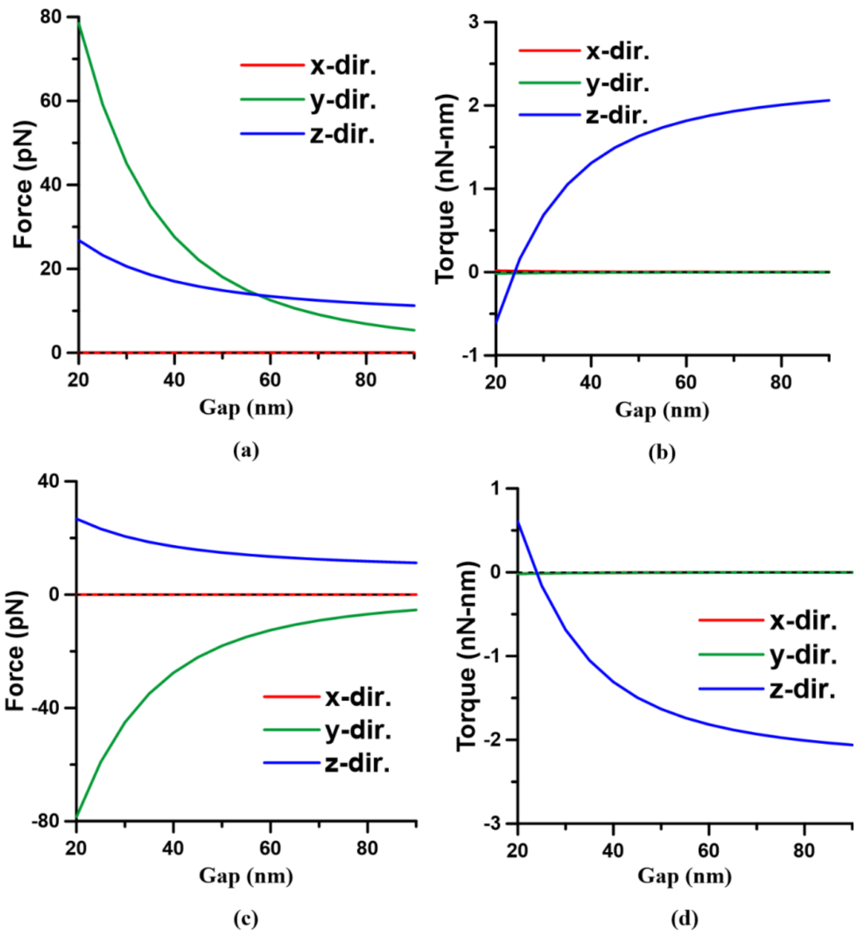


**Figure S6.** The optical (a) forces and (b) torques on the NR of *I* versus gap *d*, and (c) forces and (d) torques on the NR of *II* for the case of Fig. S1(b) with $\phi$= 45$^{\text{o}}$ at $\lambda$= 800 nm. The outcome is the repulsion.

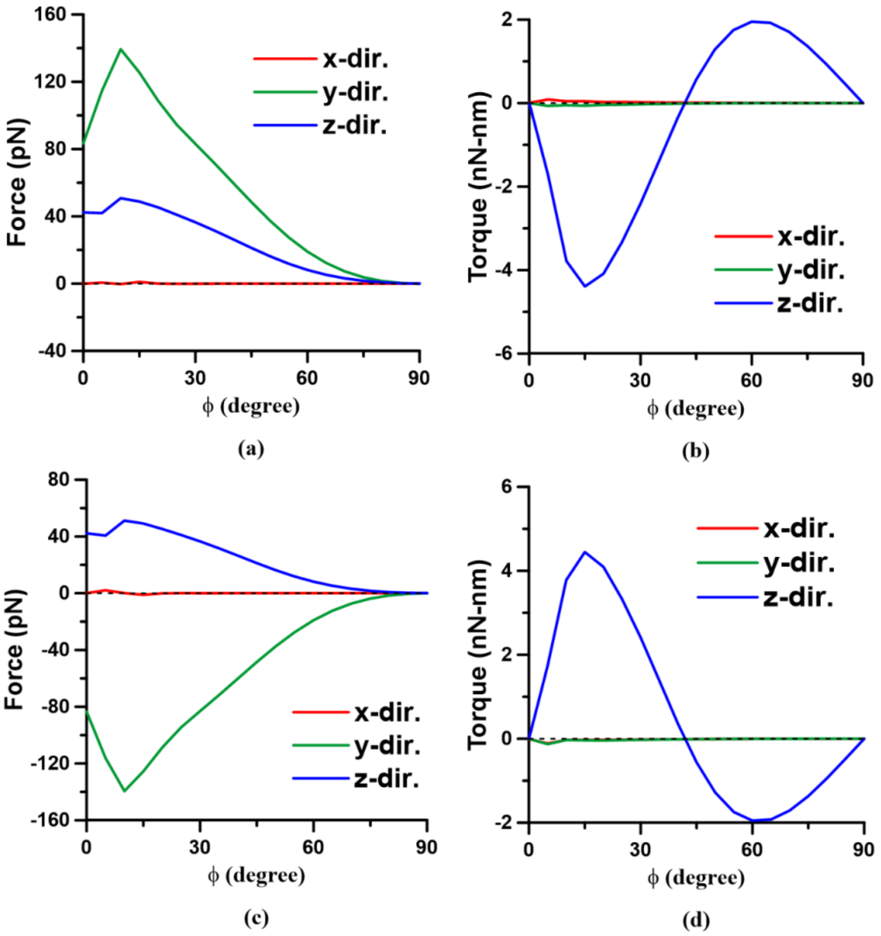


**Figure S7.** The optical (a) forces and (b) torques on the NR of *I* versus angle $\phi$, and (c) forces and (d) torques on the NR of *II* for the case of Fig. S1(b) with $d$= 20 nm at $\lambda$= 800 nm. The outcome is the repulsion.

For the case of Fig. S1(a) at $\lambda$= 1064 nm, the optical forces of $x$ component exerted on both NRs perform the attraction ($F_x^I \le 0$, $F_x^{II} \ge 0$), as shown in Figs. S8 and S9. In addition, the $y$-component optical torques make these NRs parallel to the $x$ axis ($M_y^I \ge 0$, $M_y^{II} \le 0$). Consequently, the two gold NRs can coalesce with one another in the end-to-end manner for the initial postures. However, after the coalescence, the new-formed NR of AR= 8 will be aligned by the optical torque to be perpendicular to the polarization of light.

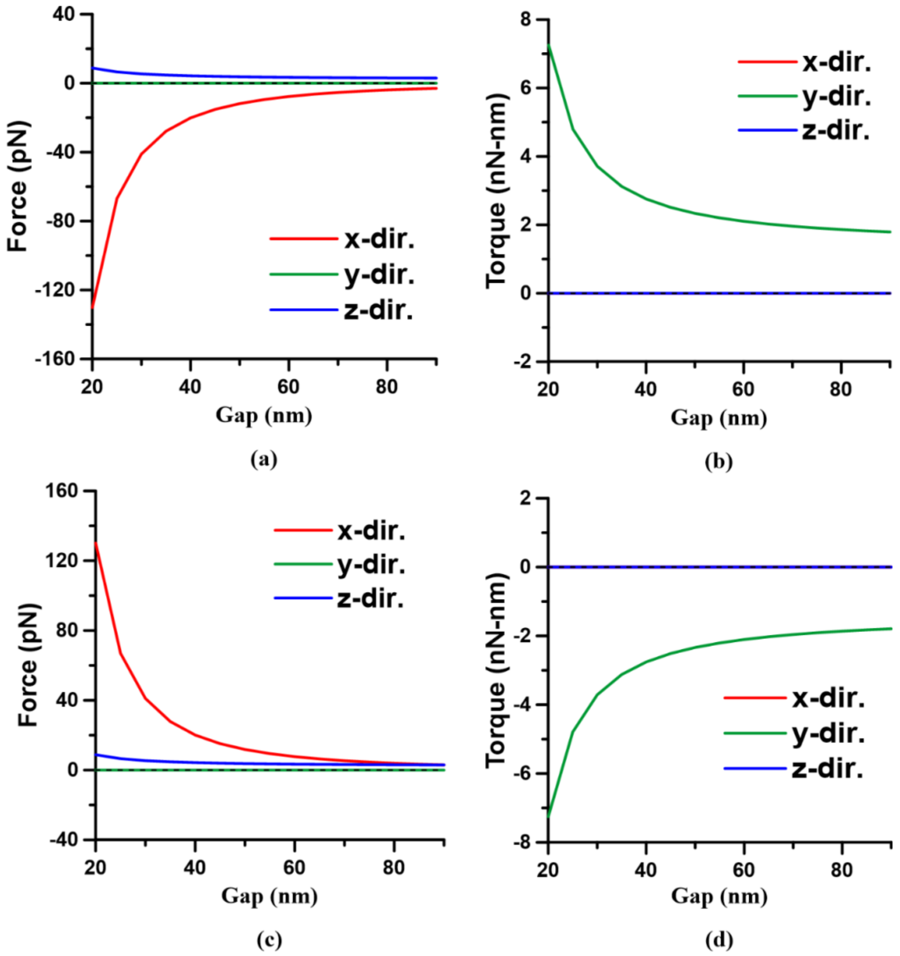


**Figure S8.** The optical (a) forces and (b) torques on the NR of $I$ versus gap $d$, and (c) forces and (d) torques on the NR of $II$ for the case of Fig. S1(a) with $\theta$= 45$^{\circ}$ at $\lambda$= 1064 nm. The outcome is the end-to-end coalescence.

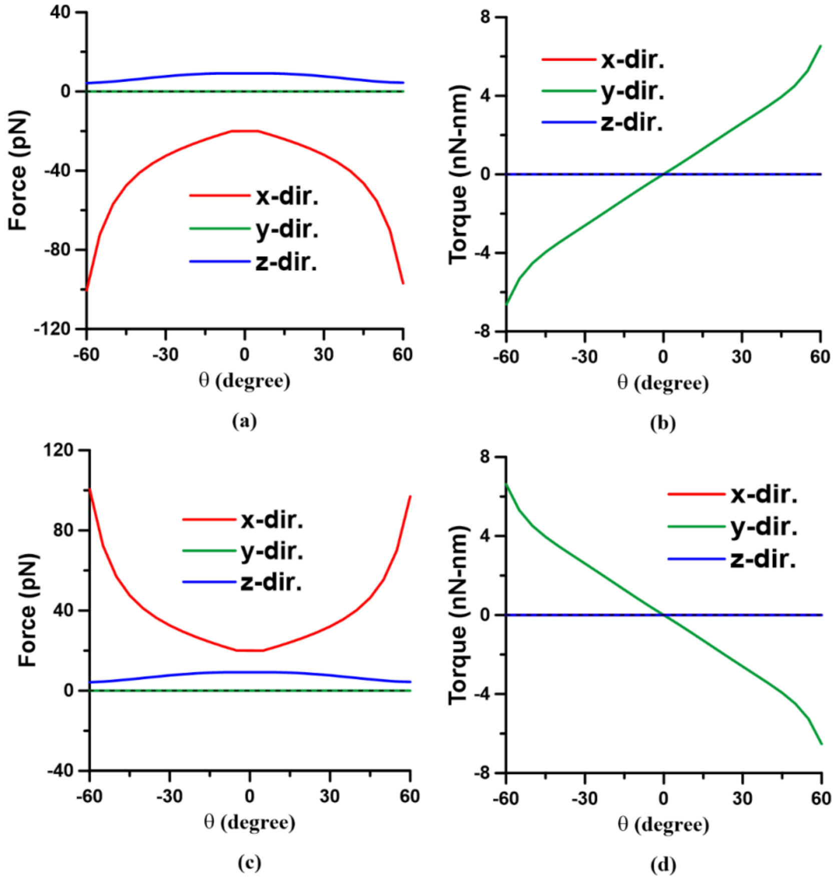


**Figure S9.** The optical (a) forces and (b) torques on the NR of *I* versus angle $\theta$, and (c) forces and (d) torques on the NR of *II* for the case of Fig. S1(a) with $d$= 20 nm at $\lambda$= 1064 nm. The outcome is the end-to-end coalescence.

For the case of Fig. S1(b) at $\lambda$= 1064 nm, the optical forces of $y$ component on both NRs, as shown in Figs. S10 and S11, perform the repulsion ($F_y^I \geq 0$, $F_y^{II} \leq 0$). Hence, the two NRs cannot coalesce with one another for the initial postures.

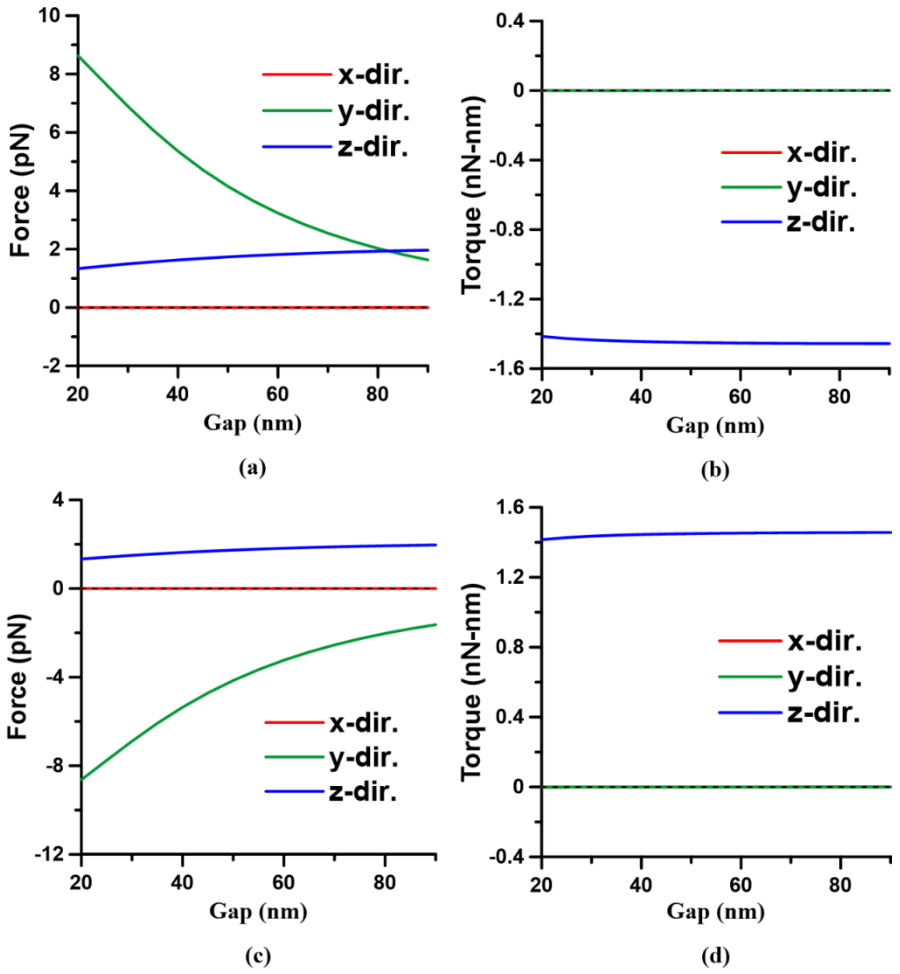


**Figure S10.** The optical (a) forces and (b) torques on the NR of *I* versus gap $d$, and (c) forces and (d) torques on the NR of *II* for the case of Fig. S1(b) with $\phi$= 45° at $\lambda$= 1064 nm. The outcome is the repulsion.

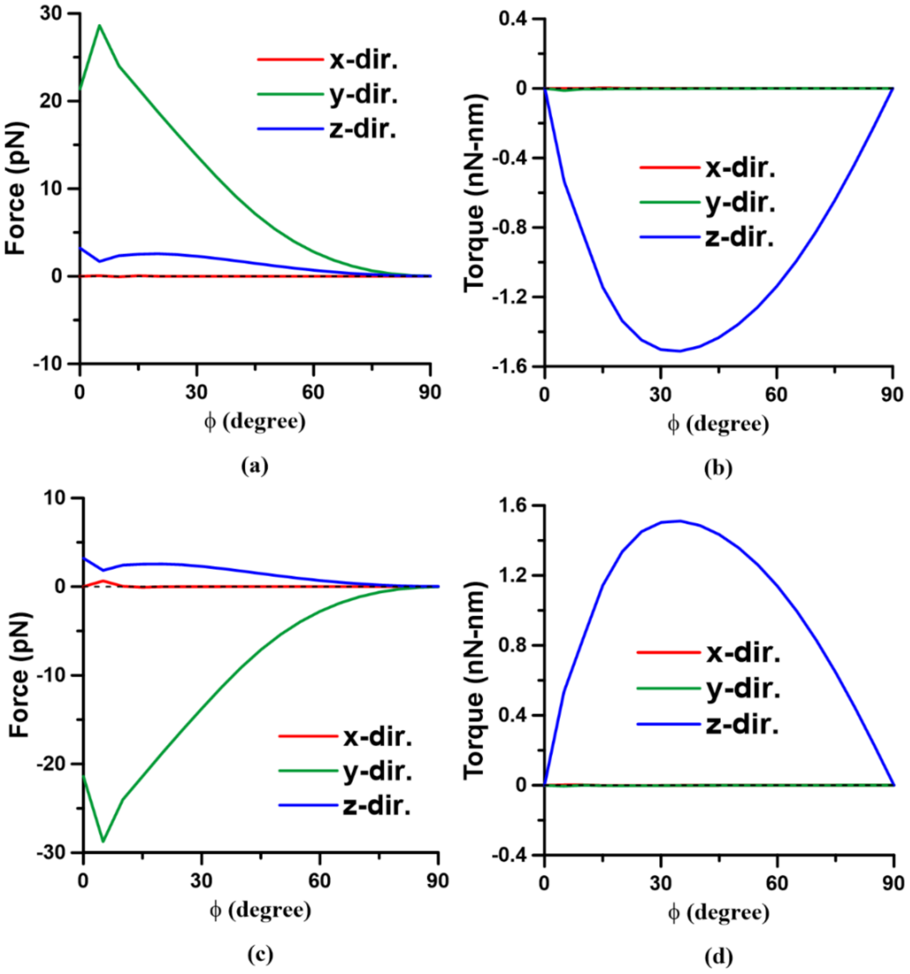


**Figure S11.** The optical (a) forces and (b) torques on the NR of *I* versus angle $\phi$, and (c) forces and (d) torques on the NR of *II* for the case of Fig. S1(b) with $d$= 20 nm at $\lambda$= 1064 nm. The outcome is the repulsion.

For the case of Fig. S1(a) at $\lambda$= 1700 nm, the $x$-component optical forces exerted on both NRs perform the attraction ($F_x^I \le 0$, $F_x^{II} \ge 0$), as shown in Figs. S12 and S13. In addition, the $y$-component optical torques make these NRs parallel to the $x$ axis ($M_y^I \ge 0$, $M_y^{II} \le 0$). Consequently, the two NRs can coalesce with one another in the end-to-end manner for the initial postures. However, after the coalescence, the new-formed NR of AR= 8 will still be aligned by the optical torque to be parallel to the polarization of light.

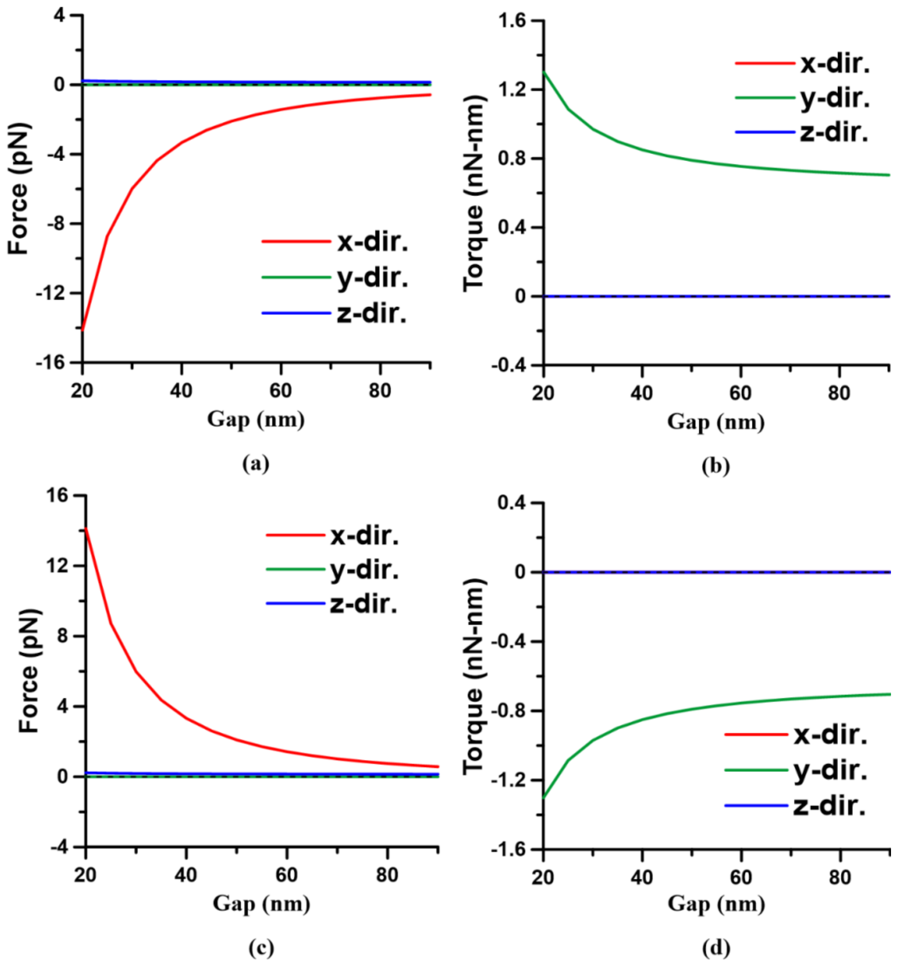


**Figure S12.** The optical (a) forces and (b) torques on the NR of $I$ versus gap $d$, and (c) forces and (d) torques on the NR of $II$ for the case of Fig. S1(a) with $\theta$= 45$^{o}$ at $\lambda$= 1700 nm. The outcome is the end-to-end coalescence.

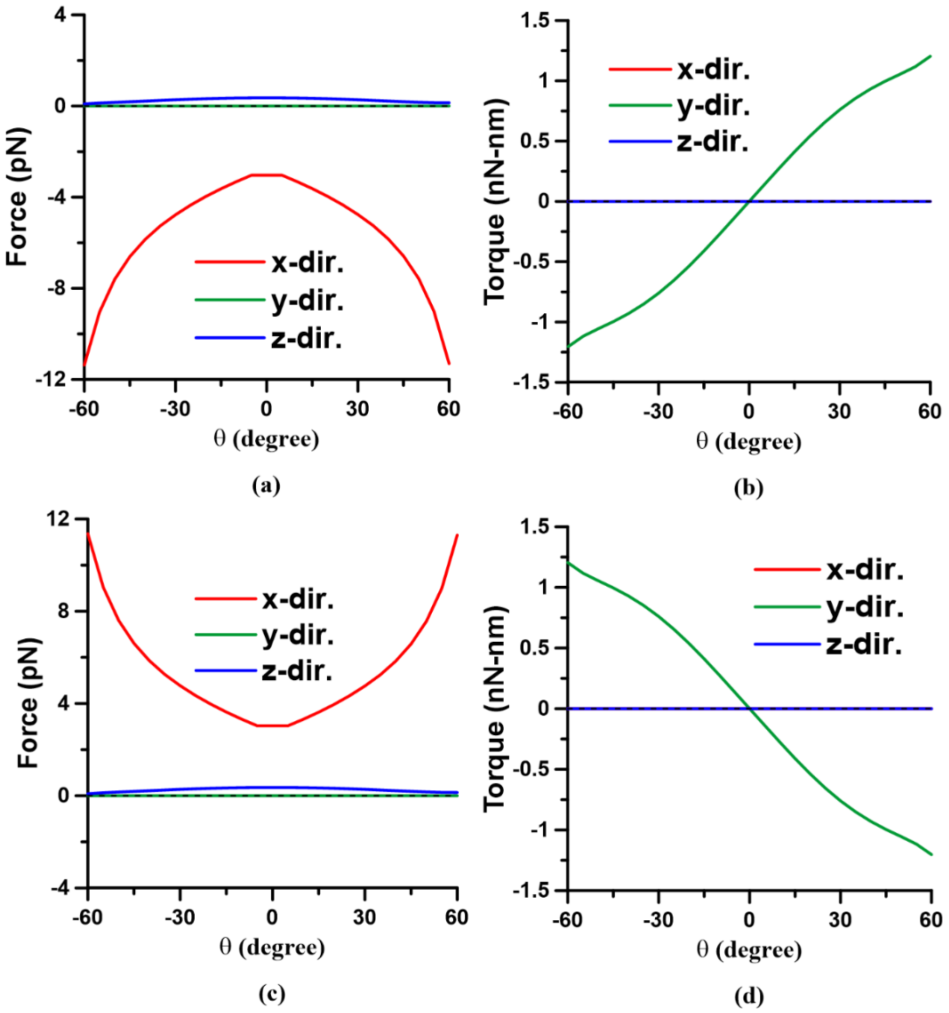


**Figure S13.** The optical (a) forces and (b) torques on the NR of *I* versus angle $\theta$, and (c) forces and (d) torques on the NR of *II* for the case of Fig. S1(a) with $d$= 20 nm at $\lambda$= 1700 nm. The outcome is the end-to-end coalescence.

For the case of Fig. S1(b) at $\lambda$= 1700 nm, the *y*-component optical forces on both NRs perform the repulsion ($F_y^I \geq 0$, $F_y^{II} \leq 0$), as shown in Figs. S14 and S15. Hence, the two NRs cannot coalesce with one another for the initial postures.

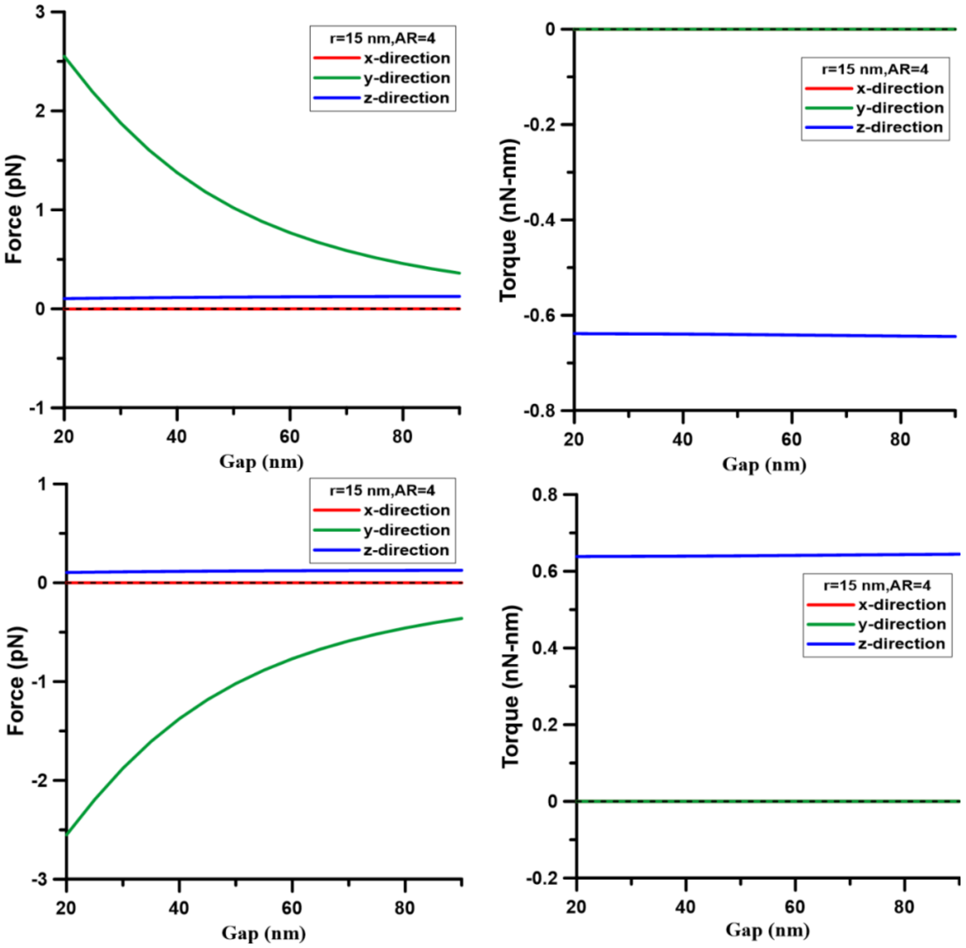


**Figure S14.** The optical (a) forces and (b) torques on the NR of *I* versus gap *d*, and (c) forces and (d) torques on the NR of *II* for the case of Fig. S1(b) with $\phi$= 45$^o$ at $\lambda$= 1700 nm. The outcome is the repulsion.

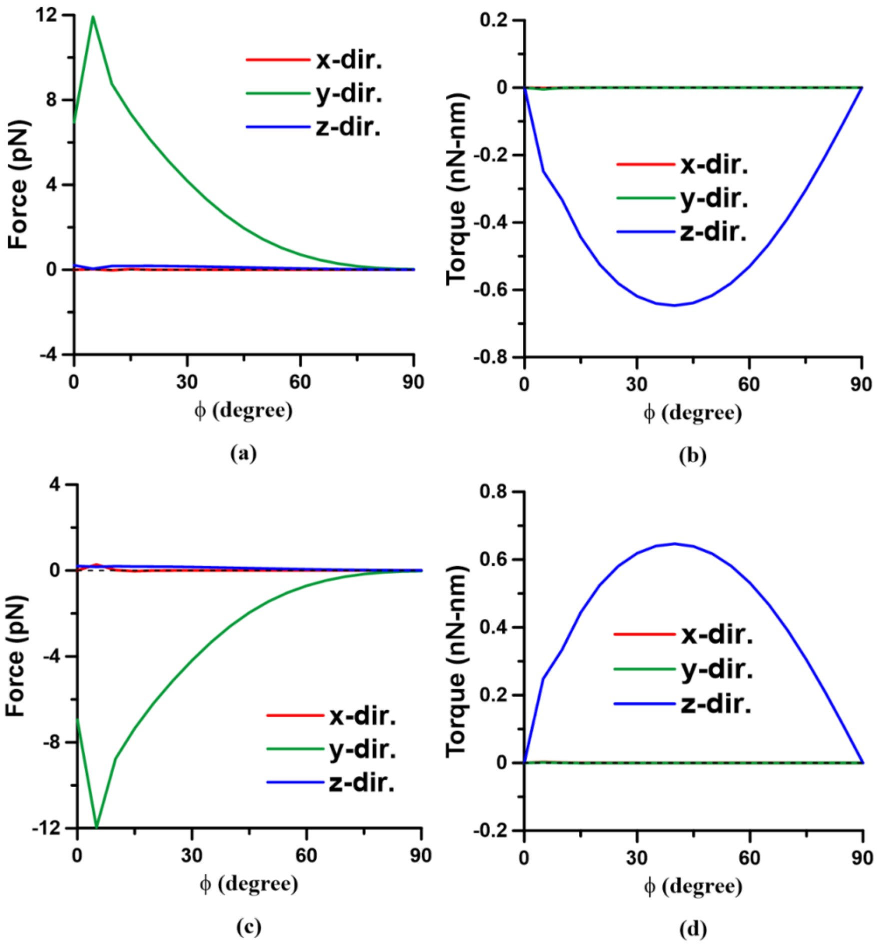


**Figure S15.** The optical (a) forces and (b) torques on the NR of *I* versus angle $\phi$, and (c) forces and (d) torques on the NR of *II* for the case of Fig. S1(b) with $d$= 20 nm at $\lambda$= 1700 nm. The outcome is the repulsion.
